# The extract of black cumin, licorice, anise, and black tea alleviates OVA-induced allergic rhinitis in mouse via balancing activity of helper T cells in lung

**DOI:** 10.1186/s13223-021-00587-6

**Published:** 2021-09-07

**Authors:** Chengsong Liao, Yangyang Han, Zhijing Chen, Huricha Baigude

**Affiliations:** 1 Xilingol Institute of Bioengineering, Xilingol Vocational College, 11 Mingantu Road, Xilinhot, 026000 Inner Mongolia People’s Republic of China; 2 School of Chemistry & Chemical Engineering, Inner Mongolia University, 235 Daxue West Road, Hohhot, 010021 Inner Mongolia People’s Republic of China

**Keywords:** Nigella sativa, Allergic rhinitis, Aqueous extract, T cell, Cytokines

## Abstract

**Background:**

A formulation of black cumin (*Nigella sativa* L.), licorice (*Glycyrrhiza glabra* L.), anise (*Pimpinella anisum* L.) and tea (*Camellia sinensis* (L.) Kuntze) (denoted BLAB tea) is traditionally used to relief allergy reaction including allergic rhinitis. However, little is known about its underlining mechanism of anti-allergic effects.

**Methods:**

To investigate the anti-allergenic mechanism of BLAB tea, we treated ovalbumin (OVA)-induced allergic rhinitis (AR) model of mice with BLAB tea, and elucidated its possible mechanism of action. Mice in the control group were treated with phosphate-buffered saline only. Subsequently, the infiltration of different inflammatory cells was measured. In addition, histopathological changes in the nasal mucosa, and the levels of allergen-specific cytokines and OVA-specific immunoglobulins were measured.

**Results:**

The aqueous extract of BLAB significantly alleviated the nasal symptoms and reduced the accumulation of inflammatory cells in the nasal mucosa and nasal lavage fluid of AR model of mice.

**Conclusion:**

The aqueous extract of BLAB induced the production of Th1 and Treg cytokines and inhibited the release of Th2 cytokines and histamine in nasal mucosa and serum of mice while decreasing the serum levels of OVA-specific IgE, IgG1, and IgG2a. These results suggest the potential of the aqueous extract of BLAB as a treatment option for allergic diseases.

**Supplementary Information:**

The online version contains supplementary material available at 10.1186/s13223-021-00587-6.

## Background

Allergic rhinitis (AR), also known as hay fever, is a chronic respiratory disease affecting 10–30% of the global population [1]. It is characterized by symptoms such as sneezing, nasal congestion, runny nose, and watery and itchy eyes; which significantly reduce the quality of the patient’s life [2]. AR is an immunoglobulin E (IgE)-mediated inflammatory response of the immune system when exposed to allergens, and involves several inflammatory cells such as eosinophils, neutrophils, mast cells, cytokines, and other inflammatory mediators [2, 3].

The two main types of helper T cells involved in AR are T-helper type 1 (Th1) and T-helper type 2 (Th2) cells. Th1-associated immune responses produce interleukin (IL)-12 and interferon (IFN)-γ [4], and Th2-associated immune responses produce IL-4, IL-5, and IL-13. Studies have shown that the pathogenesis of AR is correlated with the imbalance of Th1/Th2, resulting in the activation of Th2 cells and suppression of Th1 cells [5]. In addition, regulatory T (Treg) cells, which are known to inhibit the proliferation and activation of conventional effector T cells, have been found to play an important role in maintaining immune homeostasis [6]. Therefore, it can be hypothesized that regulation of the Th1/Th2/Treg immune balance may be an effective treatment strategy for AR and other allergic diseases [7].

Antihistamines, antileukotrienes, intranasal corticosteroids, and nasal decongestants are the commonly used drugs used for ameliorating allergic symptoms related to AR [8, 9]. However, undesirable side effects have been reported with the long-term use of these drugs, which include drug resistance and drug dependence. Therefore, drugs obtained from plant sources, which have fewer side effects and better safety profiles, have attracted increased attention in recent years [10–12]. Black cumin (Nigella sativa L.) has been known for its antioxidant, anti-inflammatory, antibacterial, antifungal, antidiabetic, analgesic, anticancer, and immunomodulatory effects [13–15]. It has been widely used as a traditional medicine for the treatment of many inflammatory and allergic diseases in countries belonging to the African, Asian, Arab, and Indian subcontinent regions [16]. Previous studies have shown that thymoquinone, a bioactive constituent of the oil of black cumin seeds, inhibited the production of IL-4, ovalbumin (OVA)-specific IgE, and reduced the expression of TNF-α and IL-1β [17]. Licorice (*Glycyrrhiza glabra* L.) is another widely used traditional medicinal herb in Asia. Glycyrrhizinic acid, a bioactive constituent of licorice root extract, is a potent inhibitor of 11-hydroxysteroid dehydrogenase and exhibits a range of corticosteroid-like activities [18]. Studies have shown that licorice exhibits many therapeutic effects, including anti-inflammatory, antioxidative, anti-allergenic, and antimicrobial properties [18–20]. Anise (*Pimpinella anisum* L.) seed is an important ingredient used in Chinese medicinal herbs. Studies have indicated that anise has antiviral, antioxidant, anthelmintic, antimicrobial, antifungal, anti-inflammatory, expectorant, and spasmolytic effects [21]. Tea (*Camellia sinensis* (L.) Kuntze), one of the most popular beverages in the world, has been used for centuries as a medicinal drink having antipyretic, anti-inflammatory, anti-allergic, antimicrobial, and antioxidative properties [22–24]. A powder mixture of a certain amount of black cumin seeds, licorice, anise seeds, and black tea (BLAB), which was also known as black cumin tea, was traditionally used for allergy relief [25]. And the allergic people usually needs to take BLAB extract continuously throughout the allergy season [25]. However, little is known about the pharmacological effect and mechanism of action of the aqueous extract of BLAB. Hence, this study aimed to investigate the anti-allergenic effects of the aqueous extract of BLAB in a specific proportion, on ovalbumin (OVA)-induced AR model and elucidate its possible mechanism of action.

## Methods

### Preparations of BLAB tea extract

The recipe of BLAB was adapted from the published book by Schleicher and Saleh [25]. Black cumin seeds were provided by the Institute of Traditional Chinese Medicine and Ethnic Medicine of Xinjiang Uygur autonomous region. Licorice, anise seeds, and black tea(Yunnan black tea) were purchased from Anhui Jiyou Chinese Medicine Decoction Pieces Co. LTD and were powdered using a pulverizer and sieved through a 100 mesh screen. Subsequently, the samples were mixed in a ratio of 3:1:1:1 to prepare BLAB samples. The BLAB powder was dissolved in distilled water with different concentrations of black cumin (100, 200, and 400 mg/mL) at a temperature of 90 ± 5 ℃ for 30 min. The mixture was centrifuged at 3000 rpm for 10 min and then the supernatant was collected by centrifuging at 3000 rpm for 10 min. The prepared aqueous extract of BLAB was stored at 4 °C for further analysis. Fresh BLAB extracts with different concentrations of black cumin (100, 200, and 400 mg/mL), were prepared for the treatment of AR model in mice. Dexamethasone (Dex, 2.5 mg/kg) was used as the positive control group.

### Experimental animals

Seventy-two specific pathogen-free five-week-old BALB/c male mice were purchased from Shanghai Jinhui Experimental Animal Co. Ltd (Shanghai, China). The mice were housed in a laminar air-flow cabinet under standard laboratory conditions at a temperature 23 ± 3 °C and relative humidity of 55 ± 10% with a 12 h dark/light cycle. The mice were allowed to acclimatize for 1 week before the start of the experiments. All procedures and experiments were approved in accordance with the guidelines of the Institutional Animal Care and Use Committee of Zhejiang province (SYXK(浙)2015–0008).

### Establishment of allergic rhinitis model and treatment

**Fig. 1:**
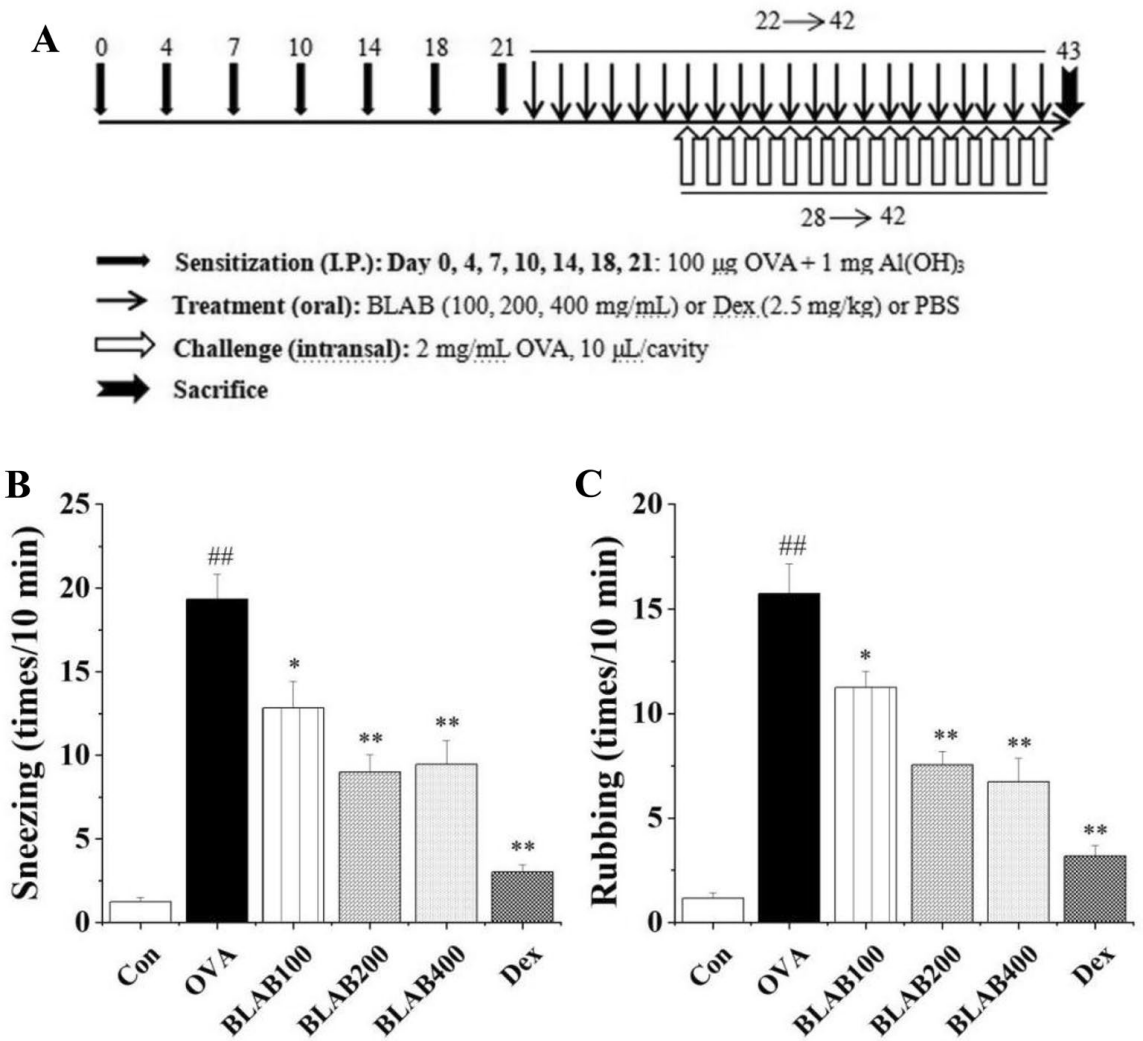
Experimental protocol for allergic rhinitis (AR) mouse model and effect of BLAB on nasal symptoms. **A** Time schedule of the OVA-induced AR mouse model and treatment with BLAB. **B** The frequency of total sneezing numbers during 10 min after the OVA challenge on day 42. **C** The frequency of total rubbing numbers during 10 min after the OVA challenge on day 42. Mice were sensitized on days 0, 4, 7, 10, 14, 18, and 21, and challenged on days 28 to 42 with OVA. Mice in the BLAB or Dex group were administered orally once daily at 100, 200, 400 mg/mL BLAB or 2.5 mg/kg dexamethasone on day 22 to 42 for 21 continuously days, respectively. The values represent the mean ± SE (n = 6/group). Significant differences at ^##^P < 0.01 compared with the control group. *P < 0.05, **P < 0.01 compared with the OVA group

Male mice were randomly divided into six groups (n = 12 per group), as follows: control, OVA, BLAB100, BLAB200, BLAB400, and Dex groups. The OVA-induced AR mouse model and treatment were established according to Piao et al.[7, 8] (Fig. 1). Briefly, OVA-induced AR mice were sensitized by intraperitoneal injection with 200 μL phosphate-buffered saline (PBS), containing 100 μg OVA (Grade V, Sigma, St. Louis, MO, USA) emulsified in 1 mg aluminum hydroxide (Thermo Scientific, Rockford, MD, USA) on days 0, 4, 7, 10, 14, 18, and 21. From day 22, the mice were orally administrated with 200 μL of BLAB solution or Dex (2.5 mg/kg) once daily, 1 h before the intranasal challenge of OVA. Mice in the control and OVA group were given 200 μL of PBS solution. One week after the last sensitization, on days 28–42, mice received an intranasal challenge with 2 mg/mL OVA, 10 μL into each nostril every day. Mice were sacrificed 24 h after the last OVA challenge for further analysis.

### Measurement of allergic symptoms

After each nostril was challenged with 10 μL OVA (2 mg/ml), the mice were placed into observation cages for evaluation of nasal symptoms. The frequencies of nasal rubbing and sneezing were measured for 10 min, immediately after the last OVA intranasal challenge [8, 9].

### Nasal lavage fluid and blood sample collection and cell count

Twenty-four hours after the last OVA challenge, blood was obtained by removing the eyeball from anesthetized mice. Immediately, the blood samples were centrifuged at 1000 × *g* for 10 min at 4 ℃ to obtain the serum, and stored at -80 ℃ for further analysis. Next, nasal lavage fluid (NALF) was collected according to the method described by Bui et al. [26]. Briefly, a catheter was inserted in the direction of the upper airway via the partially resected trachea into the nasopharynx, and 1 mL cold PBS was used to perfuse the nasal cavity gently. The collected NALF was centrifuged at 1000 × *g* for 10 min at 4 ℃. The supernatant was stored at − 80 ℃ for further analysis. The cell pellets in NALF supernatant were resuspended in the same volume of cold PBS and the cell numbers were counted with a hemocytometer. NALF (150 μL) was centrifuged onto clean glass slides using a cytospin device (GMI, Inc, Ramsey, Minnesota, USA) to measure the number of differential inflammatory cells (1000 rpm, 10 min, 4 ℃). The cells were stained using Diff-Quik kit (Beijing Solarbio Science & Technology Co. Ltd, Beijing, China), according to the manufacturer’s instructions.

### Histological examination

Referring to the method of Malmhäll et al. [5, 8], after the collection of NALF, the heads of mice were removed and fixed in 10% formalin solution for 3 days, followed by decalcification in EDTA for 5 days at 25 ± 3 ℃. Before embedding in paraffin wax, the samples were dehydrated with a series of ethyl alcohol and xylene. Nasal tissues were sectioned into 5 μm thickness and stained with hematoxylin and eosin (H&E) (Sigma-Aldich, St. Louis, MO, USA) for the examination of general morphology, periodic acid-Schiff (PAS) (Beijing Solarbio Science & Technology Co. Ltd, Beijing, China) for goblet cell hyperplasia and Giemsa (Shanghai Yuanye Biotechnology Co. Ltd, Shanghai, China) for eosinophil and mast cell infiltration. The number of goblet cells, eosinophils, and mast cells were counted, and epithelial damage was analyzed in randomly selected fields under 400 × magnification.

### Measurements of Treg cells, cytokines, and histamine

Flow cytometry and the Human Regulatory T cell Staining Kit (eBioscience, USA) for Treg cell were used according to the manufacturer’s protocols [7]. The serum levels of total IgE, anti-OVA specific IgE, IgG1, and IgG2a were measured using ELISA kits (Invitrogen, San Diego, CA, USA) and serum levels of histamine were quantified using the histamine assay kit (R&D Systems Inc, USA) [8], following the manufacturers’ instructions. The levels of cytokines (IL-4, IL-5, IL-13, IFN-γ, IL-10, and IL-12) in nasal mucosa and serum [10] were quantified using cytokine quantification kits (Invitrogen, San Diego, CA, USA), according to the manufacturer’s protocols.

### Analysis of chemical composition of BLAB tea

With reference to the method reported by Hajhashemi [15] and Shiozaki [23], Gas Chromatograph Mass Spectrometer (GC–MS) measurements were performed using a Shimadzu instrument equipped with GC: Shimadzu 2010Plus, MS: MS detector QP2020, ionization for MS: electron impact ionization, mass analyzer: single quadrupole, software: GC–MS solution, library: NIST 14 s, column: SH-Rxi-5il MS, dimensions: 30 m × 0.25 mm × 0.25 µm film thickness. The program conditions were as follows: the oven start temperature was 50 ℃ (1 min), the subsequent gradient was 25 ℃/min to 150 ℃ and then at a rate of 10 ℃/min to 300 ℃, with a final hold at 300 ℃ for 15 min and a total run time of 35 min. Carrier gas was helium, the flow rate was 1.2 mL/min, no split flow. The injection volume was 1 µL and the scan mass range was 50 m/z-500 m/z. The mass spectra in the electron ionization mode were recorded at 70 eV. The spectrum of the unknown compound was compared with the spectrum of the known compounds in the NIST 14 s library.

### Statistical analysis

Each experiment was repeated thrice. The date was expressed as means ± standard error of means, and the statistical significance of comparisons among groups was performed using one-way ANOVA, followed by Student’s test. Statistical significance was considered at the 95% confidence level (*P* < 0.05) and 99% confidence level (*P* < 0.01).

## Results

### The aqueous extract of BLAB significantly alleviated the nasal symptoms of OVA-induced AR mice

Sneezing and rubbing are commonly used to evaluate the effects of drugs on AR mice. In the present study, the frequencies of sneezing and rubbing were measured for 10 min after the last OVA intranasal challenge on day 42. It was observed that the frequencies of sneezing and rubbing of OVA group mice were significantly higher compared to that in the nontreated control group, indicating that the establishment of AR model was successful. However, the administration of aqueous extract of BLAB significantly and dose-dependently reduced the nasal symptoms compared to the OVA group. The mice in the Dex group showed a significant inhibition of nasal symptoms in OVA-induced AR mice (Fig. 1B, C).

### The aqueous extract of BLAB regulated the infiltration of inflammatory cells in NALF

**Fig. 2:**
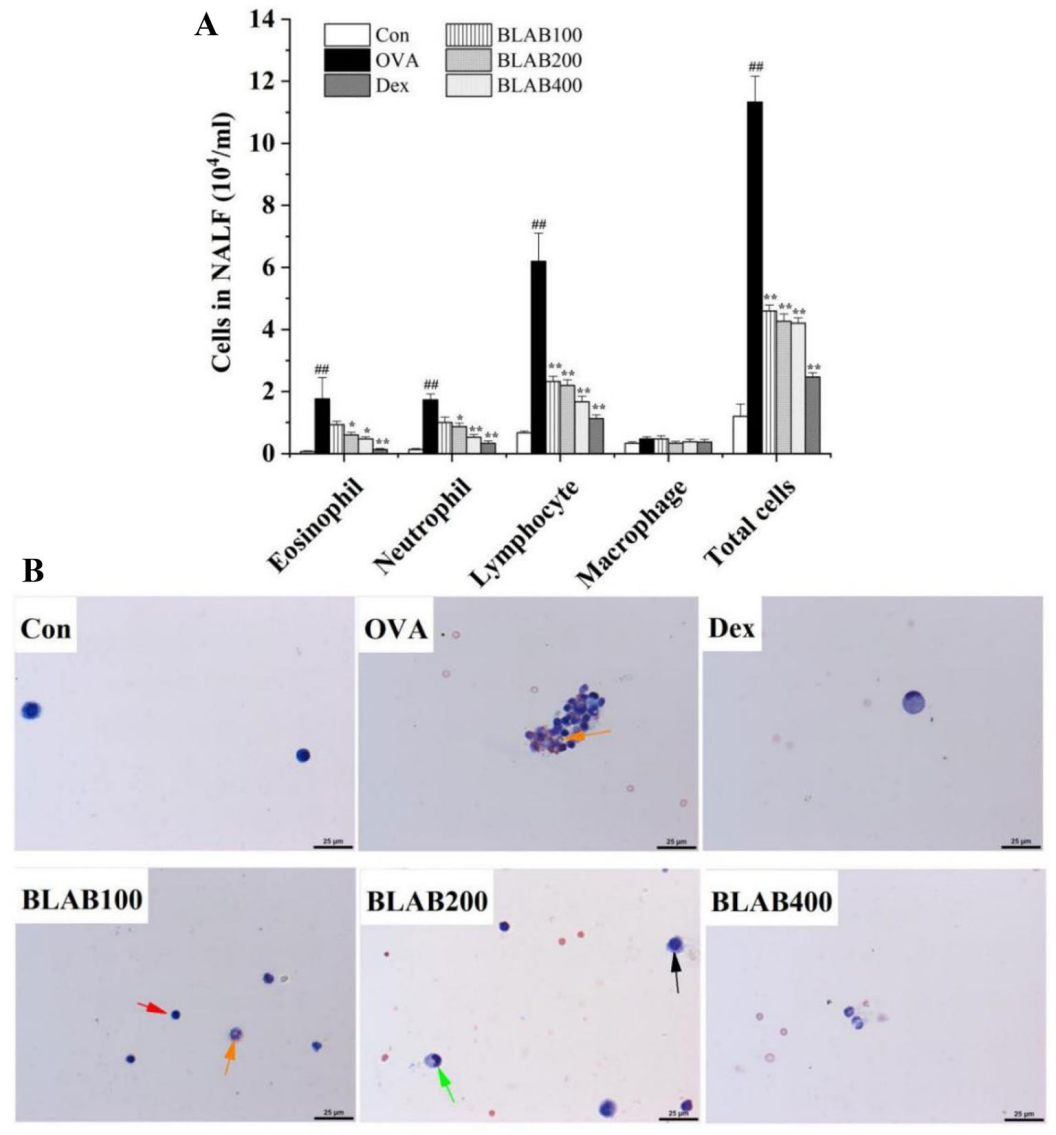
Effect of BLAB on the infiltration of differential inflammatory cells in nasal lavage fluid (NALF). **A** The number of differential cells and total cells in NALF. **B** Differential cells were stained with Diff-Quik. NALF was collected immediately after sacrifice at day 43 and the cells were isolated by cytospin. Orange, green, red, and black arrows indicate eosinophils, neutrophile, lymphocyte, and macrophages, respectively. Scale bar: 25 μm, × 400 magnification. The values represent the mean ± SE (n = 5/group). Significant differences at ^##^*P* < 0.01 compared with the control group. *P < 0.05, **P < 0.01 compared with the OVA group

The total number of inflammatory cells and number of different inflammatory cells in NALF were counted to evaluate the effect of the aqueous extract of BLAB on nasal symptoms induced by OVA. Compared to the control group, the number of differential inflammatory cells (including eosinophils, neutrophils, and lymphocytes) and the total number of inflammatory cells increased significantly in the OVA group. On the other hand, the total number of inflammatory cells and differential inflammatory cells decreased in NALF in the BLAB and Dex groups, especially in the BLAB 200 and BLAB 400 group (Fig. 2A). Results from the Diff-Quik staining showed that the number of eosinophils (orange arrow), neutrophils (green arrow), and lymphocytes (black arrow) increased significantly in the OVA group, while the numbers decreased in NALF of BLAB 200 and BLAB 400 group (Fig. 2B).

### The aqueous extract of BLAB alleviated inflammation and reduced thickness of nasal mucosa

**Fig. 3:**
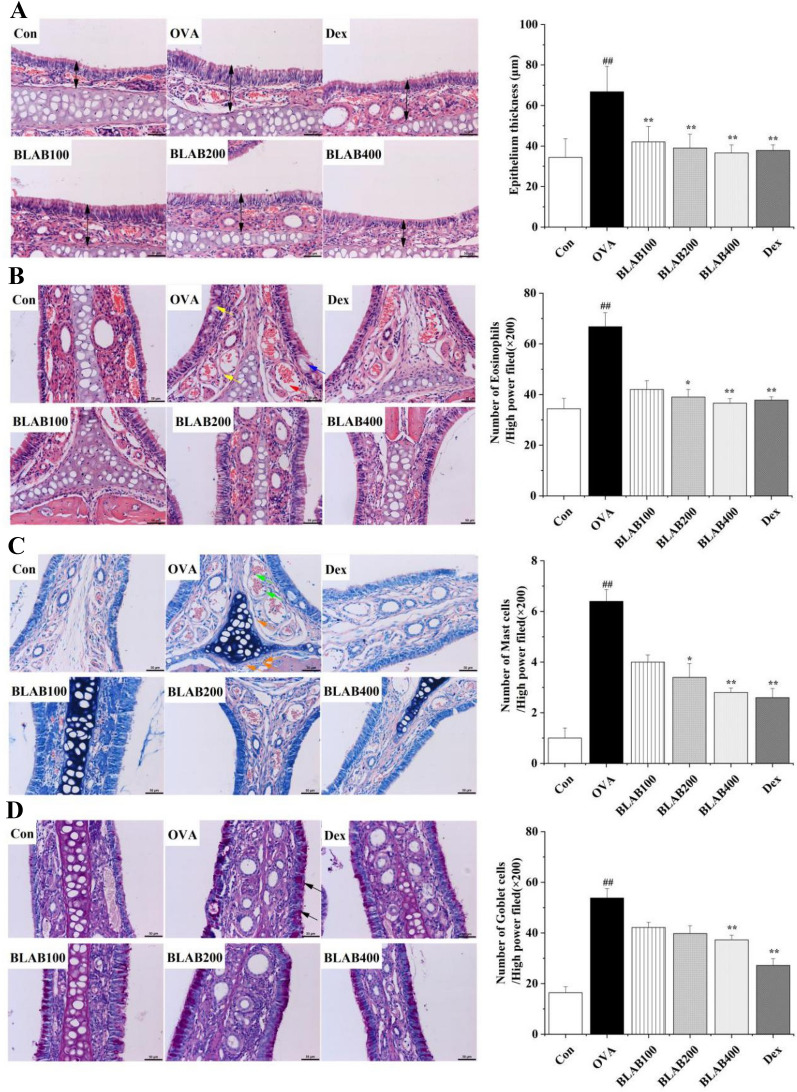
Effect of BLAB on mucosa thickness and infiltration of inflammatory cells in nasal mucosa. **A** Basic structure and epithelium thickness of mucosa. H-E staining. **B** Histopathological changes, H-E staining. **C** Infiltration of inflammatory cells. Giemsa staining. **D** Goblet cells hyperplasia, PAS staining. Blue arrows indicates damaged cilia; Red arrows indicates dilated blood vessels; Yellow arrows indicates edema in the stroma; Orange arrows indicates eosinophils; Green arrows indicates mast cells; Black arrows indicates goblet cells. Scale bar: 50 μm, × 200 magnification. Five fields of view from each group were used to count inflammatory cells. The values represent the mean ± SE (n = 5/group). Significant differences at ^##^*P* < 0.01 compared with the control group. *P < 0.05, **P < 0.01 compared with the OVA group

The nasal mucosa was stained with HE staining to determine the effect of the aqueous extract of BLAB on its histopathological features. As shown in Fig. 3A, the thickness of nasal mucosa increased significantly in the OVA group compared to the control group. Furthermore, it was observed that goblet cells accumulated in the nasal mucosa and structure of the cilia was damaged (blue arrow), the blood vessels were dilated (red arrow), and edema was observed in the stroma (yellow arrow) (Fig. 3B). Similarly, the number of goblet cells and monocyte cells was significantly higher in the OVA group than in the control group (Fig. 3C, D). In contrast, oral administration of the aqueous extract of BLAB and Dex apparently reduced the infiltration of inflammatory cells into the nasal mucosa. These data suggested that oral administration of the aqueous extract of BLAB had a protective effect on nasal mucosa structure and inflammatory cell secretion.

### Effect of the aqueous extract of BLAB on levels of Th1/Th2/Treg-associated cytokines in nasal mucosa and serum

**Fig. 4:**
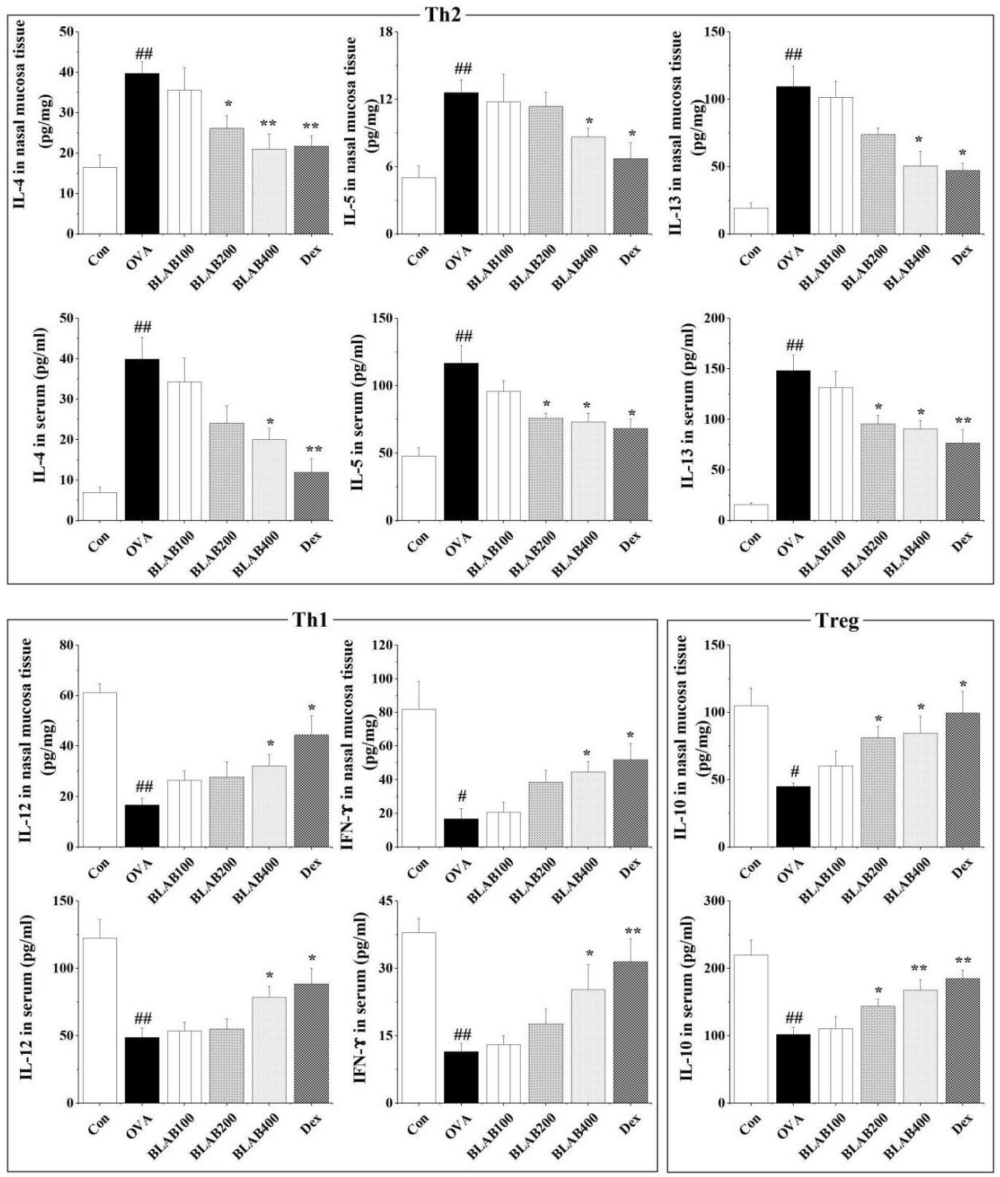
Effect of BLAB on the levels of Th1/Th2/Treg-associated cytokines in nasal mucosa and serum. The values represent the mean ± SE (n = 6/group). Significant differences at ^#^*P* < 0.05, ^##^*P* < 0.01 compared with the control group. *P < 0.05, **P < 0.01 compared with the OVA group

To examine the effect of the aqueous extract of BLAB on the modulation of T helper cell responses, the levels of Th1 (IFN-γ, IL-12), Th2 (IL-4, IL-5, IL-13), and Treg (IL-10) cytokines in nasal mucosa and serum were measured. Results showed that the levels of IL-4, IL-5, and IL-13 were significantly higher, while those of IFN-γ, IL-12, and IL-10 were significantly lower in the nasal mucosa and serum of mice in the OVA group compared to those in the control group (Fig. 4). Oral administration of the aqueous extract of BLAB notably decreased the levels of IL-4, IL-5, and IL-13 in a dose-dependent manner; both in the nasal mucosa and serum compared to the OVA group. In contrast, the levels of IFN-γ, IL-12, and IL-10 were significantly elevated (in a dose-independent manner) in the nasal mucosa and serum of mice in the BLAB group compared to the OVA group. Especially, the above indexes (IL-4, IL-5, IL-13, IFN-γ, IL-12, and IL-10) of 400 mg/kg dose BLAB treatment had statistically significant differences from those of the OVA group (Fig. 4). Thus, the results suggested that the aqueous extract of BLAB enhanced the Th1 and Treg responses and suppressed the Th2 response in nasal mucosa and serum, thus balancing the Th1/Th2/Treg response to repair abnormal allergic immune response.

### Effect of the aqueous extract of BLAB on Treg cells

**Fig. 5:**
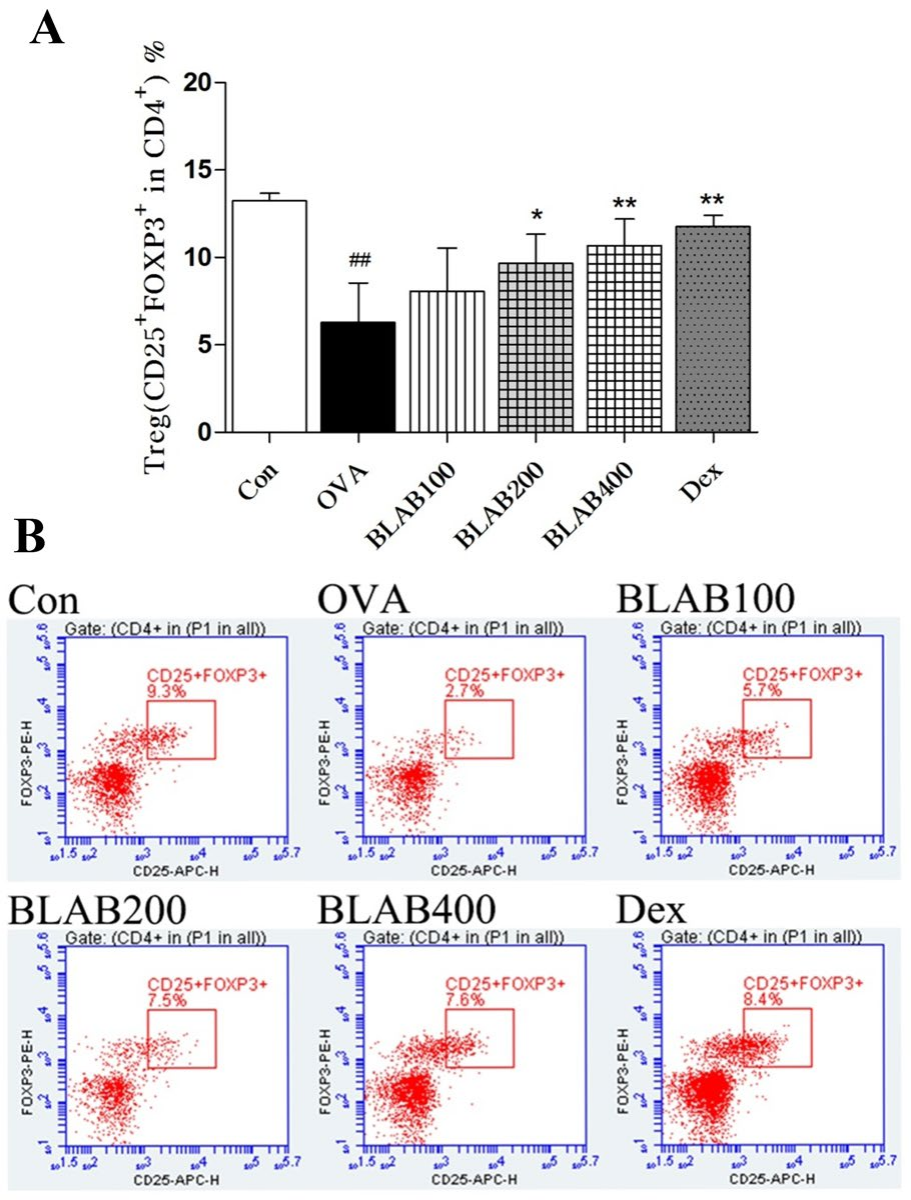
Effect of BLAB on the Treg cells. **A** Tregs levels (CD25 + FOXP3 in CD4). **B** Flow analysis of CD4/Foxp3 levels.The values represent the mean ± SE (n = 6/group). Significant differences at ^##^*P* < 0.01 compared with the control group. *P < 0.05, **P < 0.01 compared with the OVA group

To identify the source of the changes in serum IL-10, Treg levels were tested and CD4/Foxp3 levels were measured by flow cytometry (Fig. 5). As showed in Fig. 5, Treg and and CD4/Foxp3 levels were significantly lower in the OVA group compared to the control group. Administration of the aqueous extract of BLAB increased the Treg and and CD4/Foxp3 levels, especially at a dose of 200 and 400 mg/kg (Fig. 5A, B).

### The aqueous extract of BLAB regulated the levels of histamine, total IgE and OVA-specific immunoglobulins in serum

**Fig. 6:**
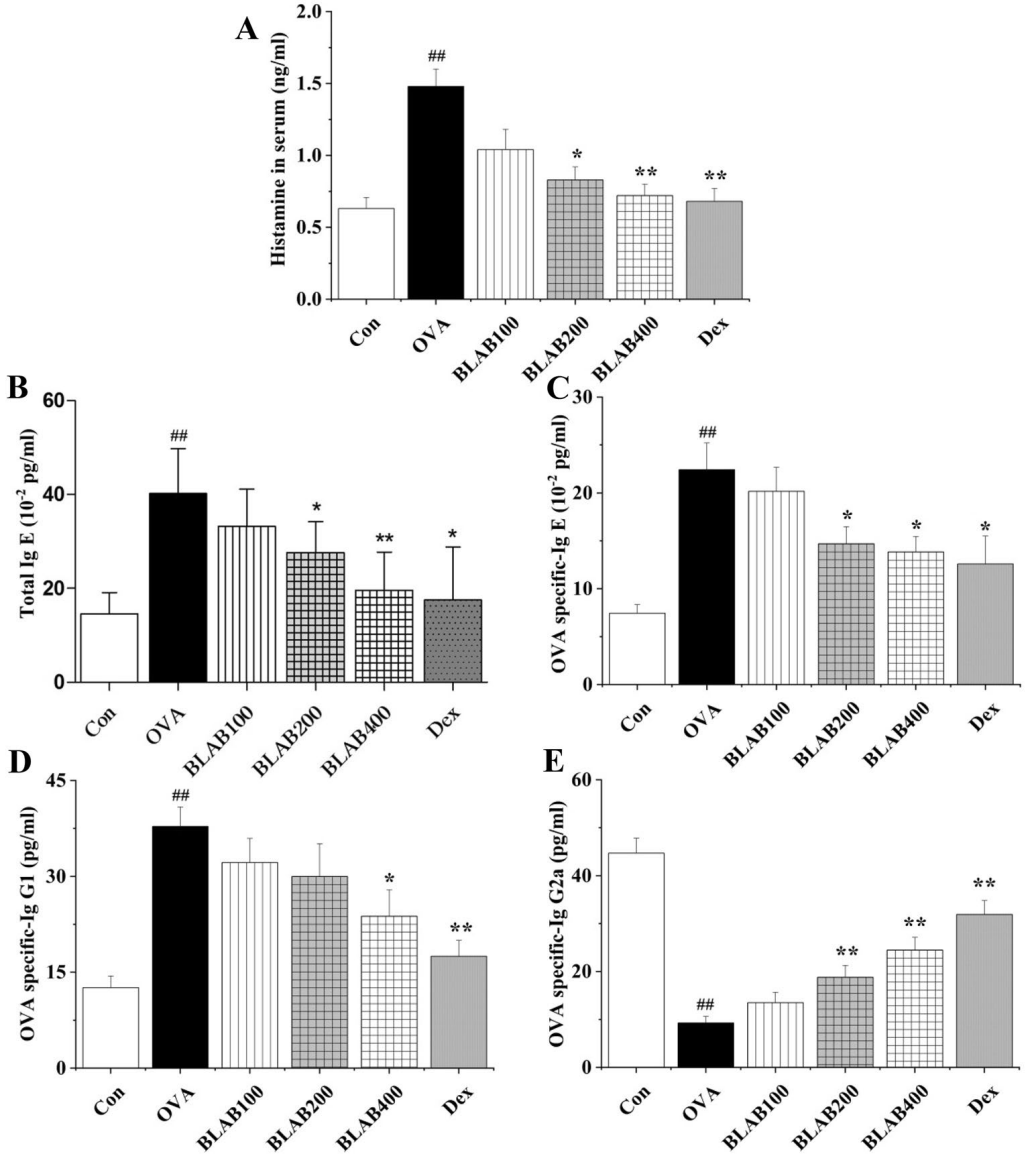
Effect of BLAB on the levels of histamine, total IgE and OVA-specific immunoglobulins in serum. **A** Histamine. **B** total IgE. **C** OVA-specific Ig E. **D** OVA-specific Ig G1. **E** OVA-specific Ig G2a. The values represent the mean ± SE (n = 6/group). Significant differences at ^##^*P* < 0.01 compared with the control group. *P < 0.05, **P < 0.01 compared with the OVA group

To evaluate the effect of the aqueous extract of BLAB on allergic inflammatory responses, the levels of histamine, total IgE, and OVA-specific immunoglobulins (IgE, IgG1, and IgG2a) in serum were detected. The levels of all inflammatory markers were significantly difference in the OVA group compared to the control group. Administration of the aqueous extract of BLAB decreased the levels of histamine, total IgE, OVA-specific IgE, and OVA-specific IgG1 in serum, especially at a dose of 400 mg/kg (Fig. 6A–D). However, as shown in Fig. 6E, the levels of OVA-specific IgG2a increased significantly in the BLAB group, in a dose-dependent manner, when compared to the OVA group.

### Analysis and identification of chemicals in the aqueous extract of BLAB

**Fig. 7:**
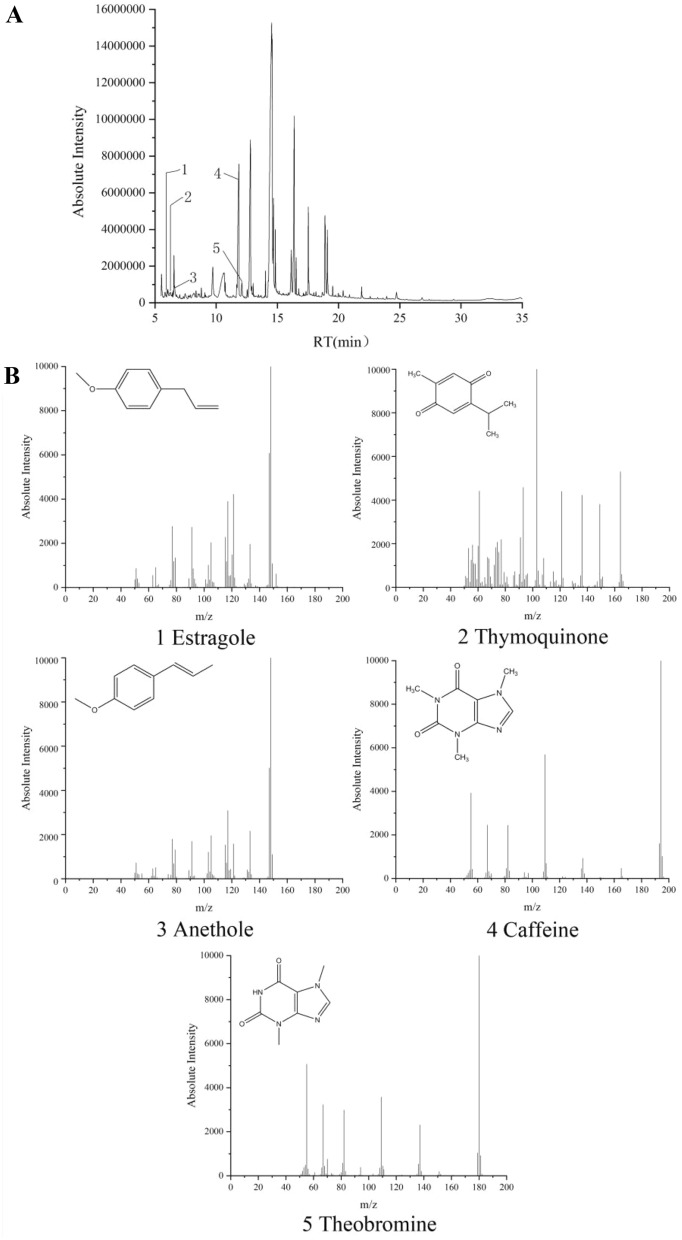
Analysis of BLAB chemicals by GC–MS. **A** The total ion flow diagram of the BLAB solution. **B** Mass spectrogram and chemical structure formula of characteristic ions in BLAB solution, 1: Estragole; 2: Thymoquinone; 3: Anethole; 4: Caffeine; 5: Theobromine

The aqueous extract of BLAB was freeze-dried to remove distilled water and then redissolved in ethanol. Analysis by GC–MS revealed multiple components in the extract, which were established via contrasting the molecular formula and fragmentation patterns with documented data in the literature. A total of 44 compounds were identified (Fig. 7, Table 1). Thymoquinone, estragole, anethole, caffeine, and theobromine, which were the bioactive components of black cumin, anise seeds, and black tea, respectively, were identified and included in Table 1. The biomarkers of licorice, glycyrrhizic acid, and liquiritin were also detected by UPLC-QTof-MS in the aqueous extract of BLAB (Additional file 1: Fig. S1, Table S1).

**Table 1:** Compounds identified in the aqueous extract of BLAB by GC–MS

No	CAS number	RT(min)	Name of the compound	Molecular formula	Molecular weight	Peak area(%)
1	28,564-83-2	5.534	4H-Pyran-4-one, 2,3-dihydro-3,5-dihydroxy-6-methyl	C_6_H_8_O_4_	144	1.40
2	78-39-7	5.822	1,1,1-triethoxy-ethan	C_8_H_18_O_3_	162	1.06
3	140-67-0	5.902	Estragole	C_10_H_12_O	148	0.06
4	496-16-2	5.992	Benzofuran, 2,3-dihydro	C_8_H_8_O	120	0.43
5	67-47-0	6.045	5-Hydroxymethylfurfural	C_6_H_6_O_3_	126	1.08
6	490-91-5	6.267	Thymoquinone	C_10_H_12_O_2_	164	0.02
7	123-11-5	6.342	Benzaldehyde, 4-methoxy	C_8_H_8_O_2_	136	0.08
8	42,786-06-1	6.419	4H-1,2,4-Triazol-3-amine, 4-ethyl	C_4_H_8_N_4_	112	0.36
9	540-97-6	6.501	Cyclohexasiloxane, dodecamethyl	C_12_H_36_O_6_Si_6_	444	0.30
10	104-46-1	6.555	Anethole	C_10_H_12_O	148	1.62
11	–	6.605	Succinic acid, nonyl 4-tert-butylphenyl ester	C_23_H_36_O_4_	376	0.20
12	–	6.675	7,7,9,9-Tetramethyl-3,6,8,10,13-pentaoxa-7,9-disilapentadecane	C_12_H_30_O_5_Si_2_	310	0.03
13	1450-72-2	6.750	Ethanone, 1-(2-hydroxy-5-methylphenyl)	C_9_H_10_O_2_	150	0.10
14	6236-09-5	7.024	Citramalic acid	C_5_H_8_O_5_	148	0.19
15	122-84-9	7.322	2-Propanone, 1-(4-methoxyphenyl)	C_10_H_12_O_2_	164	0.16
16	87-66-1	7.472	1,2,3-Benzenetriol	C_6_H_6_O_3_	126	1.18
17	475-20-7	7.724	Longifolene	C_15_H_24_	204	0.06
18	107-50-6	7.884	Cycloheptasiloxane, tetradecamethyl	C_14_H_42_O_7_Si_7_	518	0.13
19	96–76-4	8.349	2,4-Di-tert-butylphenol	C_14_H_22_O	206	0.75
20	2217-60-9	8.790	p-Cymene-2,5-diol	C_10_H_14_O_2_	166	0.82
21	51,410-48-1	9.072	1-(4-Methoxyphenyl)propane-1,2-diol	C_10_H_14_O_3_	182	0.80
22	501-97-3	9.728	Benzenepropanoic acid, 4-hydroxy	C_9_H_10_O_3_	166	5.07
23	36,256-85-6	10.640	D-Fructose, 3-O-methyl	C_7_H_14_O_6_	194	3.15
24	544-63-8	10.738	Tetradecanoic acid	C_14_H_28_O_2_	228	0.23
25	1120-16-7	10.815	Dodecanamide	C_12_H_25_NO	199	0.11
26	556-71-8	10.921	Cyclononasiloxane, octadecamethyl	C_18_H_54_O_9_Si_9_	666	0.07
27	58-08-2	11.851	Caffeine	C_8_H_10_N_4_O_2_	194	23.39
28	83-67-0	12.100	Theobromine	C_7_H_8_N_4_O_2_	180	1.77
29	373-49-9	12.532	Palmitoleic acid	C_16_H_30_O_2_	254	0.26
30	57-10-3	12.803	n-Hexadecanoic acid	C_16_H_32_O_2_	256	8.70
31	628-97-7	13.019	Hexadecanoic acid, ethyl ester	C_18_H_36_O_2_	284	0.50
32	20,675-96-1	13.081	trans-Sinapyl alcohol	C_11_H_14_O_4_	210	0.05
33	112-80-1	13.454	Oleic Acid	C_18_H_34_O_2_	112	0.08
34	–	14.026	9-Undecenal, 2,10-dimethyl	C_13_H_24_O	196	0.48
35	80,600-76-0	14.534	(Z)-18-Octadec-9-enolide	C_18_H_32_O_2_	280	28.27
36	60-33-3	15.139	9,12-Octadecadienoic acid (Z,Z)	C_18_H_32_O_2_	280	0.06
37	589-68-4	15.830	Tetradecanoic acid, 2-hydroxy-1-(hydroxymethyl)ethyl ester	C_17_H_34_O_4_	302	0.01
38	7370-49-2	16.124	cis-13,16-Docasadienoic acid	C_22_H_40_O_2_	336	1.34
39	301-02-0	16.372	9-Octadecenamide	C_18_H_35_NO	281	13.52
40	119-47-1	16.748	Phenol, 2,2'-methylenebis[6-(1,1-dimethylethyl)-4-methyl	C_23_H_32_O_2_	340	0.15
41	556-71-8	17.129	Cyclononasiloxane, octadecamethyl	C_18_H_54_O_9_Si_9_	666	0.08
42	23,470-00-0	17.523	Hexadecanoic acid, 2-hydroxy-1-(hydroxymethyl)ethyl ester	C_19_H_38_O_4_	330	1.70
43	1989-52-2	18.765	3β-tetradecanoyloxycholest-5-ene	C_41_H_72_O_2_	596	0.01
44	83-47-6	24.751	Gamma.-Sitosterol	C_29_H_50_O	414	0.15

“–” represents the CAS number of the compound was unknown

## Discussion

In this study, we investigated the effect of the aqueous extract of BLAB on OVA-induced AR model. The results showed that the aqueous extract of BLAB had a significant effect on AR through the inhibition of infiltration of differential inflammatory cells from nasal mucosa to NALF, regulation of the levels of Th1/Th2/Treg-associated cytokines in nasal mucosa and serum, and adjustment of the balance between histamine, total IgE and the OVA-specific immunoglobulins in the serum.

Infiltration of differential inflammatory cells is a typical feature of allergic diseases [2]. The accumulation of eosinophilic cells triggers regulatory factors, which in turn induce epidermal cell damage, resulting in thickening and swelling of the nasal mucosa [26, 27]. In the present study, the inflammatory cells were examined in NALF and nasal mucosa. It was observed that oral administration of the aqueous extract of BLAB dramatically decreased the number of eosinophils, neutrophils, and lymphocytes in NALF. Similarly, the number of eosinophils, mast cells, and goblet cells in the BLAB group decreased significantly when compared to the OVA-group, which effectively alleviated nasal mucosa thickening. These results suggest that the aqueous extract of BLAB could prevent the secretion of inflammatory cells from nasal mucosa layers into NALF and help in maintaining normal nasal mucosa in the AR model of mice.

Excessive activation of Th2-associated cytokines is a common response observed in airway-related diseases, such as AR and asthma [4]. A study reported an increase in the levels of Th2-associated cytokines (which are inhibited by T cells) in AR patients [28]. Increase in the levels of Th2 cytokines may generate more inflammatory cells in the nasal mucosa and NALF, thereby exacerbating airway hyperresponsiveness [29, 30]. Furthermore, up-regulated expression of Th2-associated cytokines, such as IL-4, IL-5, and IL-13 may reduce the levels and activity of Th1 cytokines (IFN-γ, IL-12), thus resulting in the imbalance of Th1/Th2 [11, 29]. Recently, Treg cells, a unique subpopulation of CD4 + T cells, which inhibit T cell proliferation and autoimmune responses, were shown to regulate Th1/Th2 balance [31, 32]. In this study, the aqueous extract of BLAB clearly decreased the infiltration of inflammatory cells, including eosinophils, neutrophils, and lymphocytes in NALF and nasal mucosa (Figs. 2, 3). This may be attributed to the expression of IFN-γ and IL-12 cytokines, supported by Th1 response and suppression of Th2 cytokines expression in the nasal mucosa (Fig. 4). In addition, IL-10 might be a cytokine of Th2, and the levels of IL-10 were significantly increased after BLAB treatment compared to the OVA group.It may promotes the humoral immunity of Th2 cells and enhances the immune protection; while IL-10 inhibits IFN-γ expressed in Th1 cells, which can reduce the effect of antigenic immunity, and relieve the symptoms of allergic rhinitis. These results provide valuable evidence regarding the important role of the aqueous extract of BLAB in regulating the Th1/Th2/Treg balance.

Previous studies have shown that several immunoglobulin antibodies, including IgE, IgG1, and IgG2a, are implicated in B-cell immune responses controlled by cytokines from helper T cells [33]. Regulatory T cells (Tregs) have an important role in regulation of immune responses, and FoxP3 was the major marker of Tregs [34]. In this study, the levels of total Ig E, OVA-specific IgE and OVA-specific IgG1 were significantly higher in the OVA group than in the control group. In contrast, the levels were lower in BLAB and Dex groups compared to the OVA group (Fig. 6). Similar results were observed for the levels of IL-4, IL-5, and IL-13 in serum (Fig. 4), thus suggesting that the aqueous extract of BLAB may downregulate Th2 immune response in serum. In contrast, the levels of Treg, FoxP3, OVA-specific IgG2a, and serum levels of IL-12, IFN-γ, and IL-10 were elevated in BLAB and Dex groups (Figs. 4, 5, 6), suggesting that the aqueous extract of BLAB may upregulate Th1 and Treg immune response. In addition, the levels of histamine one of the most potent vasoactive mediators implicated in the acute phase of immediate hypersensitivity [7, 35], which were decreased in the BLAB group compared to those in the OVA group (Fig. 5A). These results verified the effect of the aqueous extract of BLAB in restoring the Th1/Th2 /Treg balance.

In our study, BLAB was prepared from a powder mixture consisting of black cumin seeds, licorice, anise seeds, and black tea. Previous studies have proven the anti-inflammatory effects of thymoquinone (TQ), the bioactive compound in black cumin seeds, in allergic and obstructive lung diseases as well as other respiratory diseases [13, 16, 17, 36]. The compound exerts its effects by inhibiting the production of IL-4 and OVA-specific IgE and reducing eosinophil infiltration and edema in the nasal mucosa [17], which was consistent with our investigation. In contrast, TQ has no effect on the levels of IFN-γ and IL-10 [17]. However, in our study, BLAB significantly increased the levels of IFN-γ and IL-10. This could be possibly due to other compounds in black cumin or other components. GC–MS analysis identified TQ and anethole, estragole as the bioactive compounds in black cumin and anise seeds, respectively. It was also found that the bioactive components in black tea were caffeine and theobromine (Fig. 6, Table 1). These compounds have shown to have anti-inflammatory properties [21]. Glycyrrhizic acid and liquiritin were identified as the bioactive compounds in licorice by UPLC-QTof-MS (Supplemental Fig. S1, Supplemental Table S1). These compounds have also demonstrated anti-inflammatory effects [19, 20], therefore, it could be reasonably inferred that the anti-allergic inflammatory effects of the aqueous extract of BLAB may be the result of a combination of these anti-inflammatory compounds, and more experiments will be needed to confirm these findings.

## Conclusions

In conclusion, our results demonstrated that the aqueous extract of BLAB exerts anti-allergic inflammatory effects on AR murine model by suppressing the accumulation of inflammatory cells in nasal mucosa and NALF, inducing Th1 and Treg cytokines production, and inhibiting Th2 cytokine and histamine release in nasal mucosa and serum. These data suggest that the aqueous extract of BLAB may have promising potential as a treatment alternative for allergic diseases.

## Supplementary Information

**Additional file 1: Figure S1.** Analysis of the aqueous extract of BLAB chemicals by UPLC-QTof-MS.** Table S1.** Compounds identified in the aqueous extract of BLAB by UPLC-QTof-MS.

## Abbreviations

BLAB tea: A formulation of black cumin (*Nigella sativa* L.), licorice (*Glycyrrhiza glabra* L.), anise (*Pimpinella anisum* L.) and tea (*Camellia sinensis* (L.) Kuntze)
OVA: Ov-albumin
