# Supplementary material for: The extract of black cumin, licorice, anise, and black tea alleviates OVA-induced allergic rhinitis in mouse via balancing activity of helper T cells in lung
Source: Allergy Asthma Clin Immunol. 2021 Sep 7;17:87. doi: 10.1186/s13223-021-00587-6 (PMC8424864; doi:10.1186/s13223-021-00587-6)
Supplement: Supplementary file 1 — Additional file 1: Figure S1. Analysis of the aqueous extract of BLAB chemicals by UPLC-QTof-MS. Table S1. Compounds identified in the aqueous extract of BLAB by UPLC-QTof-MS. [file 13223_2021_587_MOESM1_ESM.docx]

**
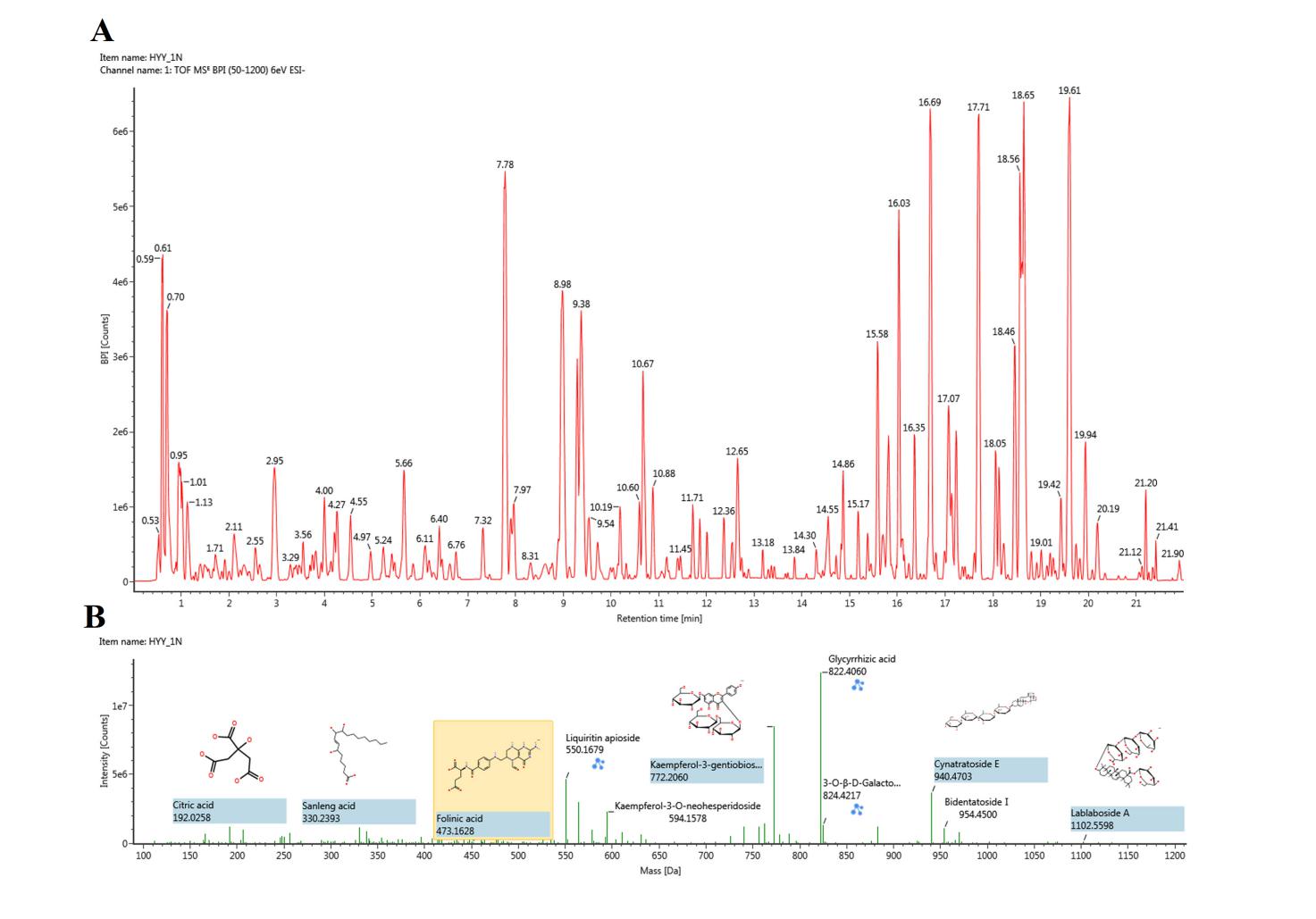
**

**Additional file 1: Figure S1.** Analysis of the aqueous extract of BLAB chemicals by UPLC-QTof-MS.

**Additional file 1: Table S1**. Compounds identified in the aqueous extract of BLAB by UPLC-QTof-MS.

| **Component name** | **Formula** | **Neutral mass (Da)** | **Observed neutral mass (Da)** | **Observed m/z** | **Mass error (mDa)** | **Observed RT (min)** | **Adducts** |
| --- | --- | --- | --- | --- | --- | --- | --- |
| Quinic acid_1 | C_7_H_12_O_6_ | 192.06339 | 192.0616 | 191.0544 | -1.7 | 0.6 | -H, +HCOO |
| Citric acid | C_6_H_8_O_7_ | 192.027 | 192.0258 | 191.0185 | -1.2 | 0.71 | -H |
| Arctic acid | - | 247.99657 | 247.9982 | 246.9909 | 1.6 | 2.12 | -H |
| 3-Hydroxyphenyl-propionic acid | - | 166.06299 | 166.0619 | 165.0546 | -1.1 | 2.95 | -H |
| 3-O-trans-Coumaroylquinic acid | - | 338.10017 | 338.0992 | 337.0919 | -1 | 5.67 | -H |
| Quercetin-3-gentiobioside-7-glucoside | - | 788.20112 | 788.2012 | 787.194 | 0.1 | 6.76 | -H |
| Kaempferol-3-gentiobioside-7-glucoside | - | 772.20621 | 772.206 | 771.1988 | -0.2 | 7.78 | -H, +HCOO |
| Apiin | C_26_H_28_O_14_ | 564.14791 | 564.1474 | 563.1401 | -0.5 | 7.96 | -H |
| Kaempferol-3,7-di-O-β-D-glucopyranoside | - | 610.15338 | 610.153 | 609.1457 | -0.4 | 8.9 | -H |
| Isoliquiritin | C_21_H_22_O_9_ | 418.12638 | 418.1251 | 417.1178 | -1.3 | 8.98 | -H |
| Patuletin-7-O-[6′′-(2-methylbutyryl)]-glucoside | - | 564.14791 | 564.1471 | 609.1453 | -0.8 | 9.3 | +HCOO |
| Quercetin-3-O-β-D-glucuronide | - | 478.07474 | 478.0739 | 477.0666 | -0.9 | 9.35 | -H |
| Liquiritin apioside | C_26_H_30_O_13_ | 550.16864 | 550.1679 | 549.1606 | -0.8 | 9.38 | -H |
| 6-Hydroxykaempferol-3-O-glucoside | - | 464.09548 | 464.0953 | 463.088 | -0.2 | 9.54 | -H |
| Sophorabioside | C_27_H_30_O_14_ | 578.16356 | 578.1635 | 577.1563 | 0 | 9.73 | -H |
| Nelumboroside B | - | 756.21129 | 756.2111 | 755.2038 | -0.2 | 10.19 | -H |
| Kaempferol-3-O-(2G-α-L-rhamnosyl)-rutinoside | - | 740.21638 | 740.2162 | 739.2089 | -0.2 | 10.6 | -H |
| Kaempferol-3-O-neohesperidoside | - | 594.15847 | 594.1578 | 593.1505 | -0.7 | 10.68 | -H |
| Populnin | C_21_H_20_O_11_ | 448.10056 | 448.0995 | 447.0923 | -1 | 10.88 | -H |
| 19-O-[β-D-Apiofuran-osyl(1→2)-β-D-gluco-pyranoyl]-3,14-dideo-xyandrographolide | - | 630.32514 | 630.325 | 675.3232 | -0.2 | 12.01 | +HCOO |
| Isoliquiritigenin | C_15_H_12_O_4_ | 256.07356 | 256.0726 | 255.0654 | -0.9 | 12.66 | -H |
| Magnolin | C_23_H_28_O_7_ | 416.1835 | 416.1856 | 415.1783 | 2.1 | 14.54 | -H |
| Cynatratoside C | - | 778.41396 | 778.4138 | 823.412 | -0.2 | 14.83 | +HCOO |
| Bidentatoside Ⅰ | - | 954.44604 | 954.45 | 999.4482 | 4 | 15.18 | +HCOO |
| 4-Hydroxy-3-butylphthalide | C_12_H_14_O_3_ | 206.09429 | 206.0929 | 251.0911 | -1.4 | 15.81 | +HCOO, -H |
| Sanleng acid | - | 330.24062 | 330.2393 | 329.2321 | -1.3 | 17.07 | -H |
| Cynatratoside E | - | 940.46678 | 940.4703 | 985.4685 | 3.5 | 18.46 | +HCOO |
| Glycyrrhizic acid | C_42_H_62_O_16_ | 822.40379 | 822.406 | 821.3987 | 2.2 | 18.63 | -H |
| Picfeltarraenin IA | C_41_H_62_O_13_ | 762.41904 | 762.4203 | 807.4185 | 1.3 | 19.43 | +HCOO |
| 3-O-β-D-Galactopyra-nosyl-(1→2)-[β-D-xylo-pyranosyl-(1→3)]-β-D-6-O-methylglucuro-nopyranosyl quillaic acid | - | 970.47734 | 970.4815 | 969.4742 | 4.1 | 19.56 | -H |
| 3-O-β-D-Galactopyra-nosyl-(1→2)-β-D-glucuronopyranosyl gypsogenin | - | 824.41944 | 824.4217 | 823.4144 | 2.3 | 20.19 | -H, +HCOO |
| Kalopanaxsaponin I | - | 882.49769 | 882.5 | 881.4928 | 2.3 | 21.2 | -H, +HCOO |
